# Activation of EGFR, HER2 and HER3 by neurotensin/neurotensin receptor 1 renders breast tumors aggressive yet highly responsive to lapatinib and metformin in mice

**DOI:** 10.18632/oncotarget.1632

**Published:** 2014-10-03

**Authors:** Sandra Dupouy, Van Kien Doan, Zherui Wu, Najat Mourra, Jin Liu, Olivier De Wever, Frédérique Penault Llorca, Anne Cayre, Amal Kouchkar, Anne Gompel, Patricia Forgez

**Affiliations:** ^1^ UMRS U938, Hôpital Saint-Antoine, Paris, France; ^2^ UMRS 1007 Université Paris Descartes 45, Paris, France; ^3^ Pathology Department Hôpital Saint-Antoine, Paris, France; ^4^ The Laboratory of Experimental Cancerology, Ghent University Hospital, Ghent, Belgium; ^5^ Pathology Department, Jean Perrin Center, Clermont Ferrand, France; ^6^ Pathology Department, Alger Pierre and Marie Curie Center, Algeria; ^7^ Gynecology Unit, Université Paris Descartes, APHP, Hôpitaux Universitaires Cochin Hôtel-Dieu Broca, Paris, France

**Keywords:** Cancer growth and metastasis, neurotensin, EGFR, HER2, HER3, EGF like ligands

## Abstract

A present challenge in breast oncology research is to identify therapeutical targets which could impact tumor progression. Neurotensin (NTS) and its high affinity receptor (NTSR1) are up regulated in 20% of breast cancers, and NTSR1 overexpression was shown to predict a poor prognosis for 5 year overall survival in invasive breast carcinomas. Interactions between NTS and NTSR1 induce pro-oncogenic biological effects associated with neoplastic processes and tumor progression. Here, we depict the cellular mechanisms activated by NTS, and contributing to breast cancer cell aggressiveness.

We show that neurotensin (NTS) and its high affinity receptor (NTSR1) contribute to the enhancement of experimental tumor growth and metastasis emergence in an experimental mice model. This effect ensued following EGFR, HER2, and HER3 over-expression and autocrine activation and was associated with an increase of metalloproteinase MMP9, HB-EGF and Neuregulin 2 in the culture media. EGFR over expression ensued in a more intense response to EGF on cellular migration and invasion. Accordingly, lapatinib, an EGFR/HER2 tyrosine kinase inhibitor, as well as metformin, reduced the tumor growth of cells overexpressing NTS and NTSR1. All cellular effects, such as adherence, migration, invasion, altered by NTS/NTSR1 were abolished by a specific NTSR1 antagonist. A strong statistical correlation between NTS-NTSR1-and HER3 (p< 0.0001) as well as NTS-NTSR1-and HER3- HER2 (p< 0.001) expression was found in human breast tumors.

Expression of NTS/NTSR1 on breast tumoral cells creates a cellular context associated with cancer aggressiveness by enhancing epidermal growth factor receptor activity. We propose the use of labeled NTS/NTSR1 complexes to enlarge the population eligible for therapy targeting HERs tyrosine kinase inhibitor or HER2 overexpression.

## INTRODUCTION

The mortality rate of breast cancer has been stabilized due to early detection and constant progress in therapy. Nevertheless, breast cancer remains the leading cause of cancer-related deaths among women in most of the western countries [1,2]. The progression of tumors to a metastatic disease is the primary cause of death in most patients and the main target of cancer research.

The human epidermal growth factor receptor (HERs) signaling pathways have been shown to largely contribute to this process. The success of therapies employing HERs immunotherapy or tyrosine kinase inhibitors, while limiting the progression of the disease and extending the disease free survival time, demonstrates the contribution of HERs at the clinical level [3]. In breast cancer, where human epidermal growth factor receptor 2 (HER2) gene amplication is detected in 25% of patients [4], a monoclonal antibody targeting HER2, trastuzumab, (Herceptin) significantly improves the survival of these patients [5]. Nevertheless, contribution of the HERs in breast cancer progression is complex, and studies have focused more specially on HER3/HER2 or HER2/EGFR dimers. HER3 and HER2 overexpression and activation driven growth and metastasis are associated with a worse prognosis [6,8]. The availability of the EGFR inhibitors, proposed to patients in association with HER2 immunotherapy, has been a real benefit [9]. For example, patients with metastatic breast cancer, which had experienced progression under trastuzumab, found a benefit from the association of trastuzumab and Pertuzumab [5], a recombinant humanized monoclonal antibody binding to the HER2 dimerization domain and preventing dimerization of HER2 with other HER receptors (HER3, HER1, and HER4) [10]. More recently, investigations have focused on autocrine HERs activation in cells lacking HER2 amplification. This cellular context generates regulatory mechanisms involved in cancer progression and is suspected to induce drug resistance. These mechanisms remain to be clarified, and the identification of factors inducing this cellular context would help to better characterize and treat breast tumor.

Amongst the factors contributing to tumor aggressiveness, neurotensin (NTS) and its cognate high-affinity receptor (NTSR1) have been shown to contribute to breast cancer progression [11,12]. High proportion of pro NTS in the blood is also correlated with a higher risk of breast cancer [13]. NTS and NTSR1 are concomitantly overexpressed in patients with breast cancer [14]. The deregulated expression profile of the NTSR1 was correlated with negative prognostic parameters such as tumor size, the number of invaded lymph nodes, Scarff, Bloom and Richardson's histoprognostic grade, and patient mortality [14]. In corroborating clinical experiments, a direct effect of the neurotensinergic system on breast tumor growth in mice, and the anti-apoptotic property of NTS on breast cancer cellular models was also shown [15,16].

Under physiology, NTS, a 13 amino acids peptide, is mainly localized in endocrine N-cells of the gastrointestinal tract, where it regulates various functions such as inhibition of gut motility and gastric acid secretions, stimulation of pancreatic and biliary secretions and facilitation of fatty acids intake [17,19]. The effects of NTS are mediated by three subtypes of receptor. NTSR1 and NTSR2 belong to the class A GPCRs [20] and exhibit high (sub-nanomolar) and low (nanomolar) affinity for NTS, respectively. NTSR3 or gp/95/sortilin is a single transmembrane domain receptor. The NTSR1 often overexpressed in tumors, activates many physiological effectors participating in cell proliferation, migration, invasion and remodeling of the actin cytoskeleton, like MAPK, focal adhesion kinase (FAK), and RhoGTPases. NTSR1 activation also transactivates the EGFR receptor in colonic, prostatic and pancreatic cancer cell lines [21,23].

We propose that a cross-talk occurs between neurotensinergic and the HER pathways in breast tumors which effects breast cancer progression. In this report, we analyzed this hypothesis, and showed that a NTS autocrine signaling loop dramatically accelerates the tumor growth and the metastatic progression of a poorly aggressive breast adenocarcinoma cell line. We analyzed the oncogenic cellular effects enhanced by NTS, and showed a possible synergic interaction between EGF and NTS, and observed a complete remodeling of HERs basal and activated profiles in cells under the constant stimulation by NTS.

## RESULTS

### The NTS-NTSR1 complex enhances tumor growth and metastasis emergence from breast experimental tumor

**Figure 1:**
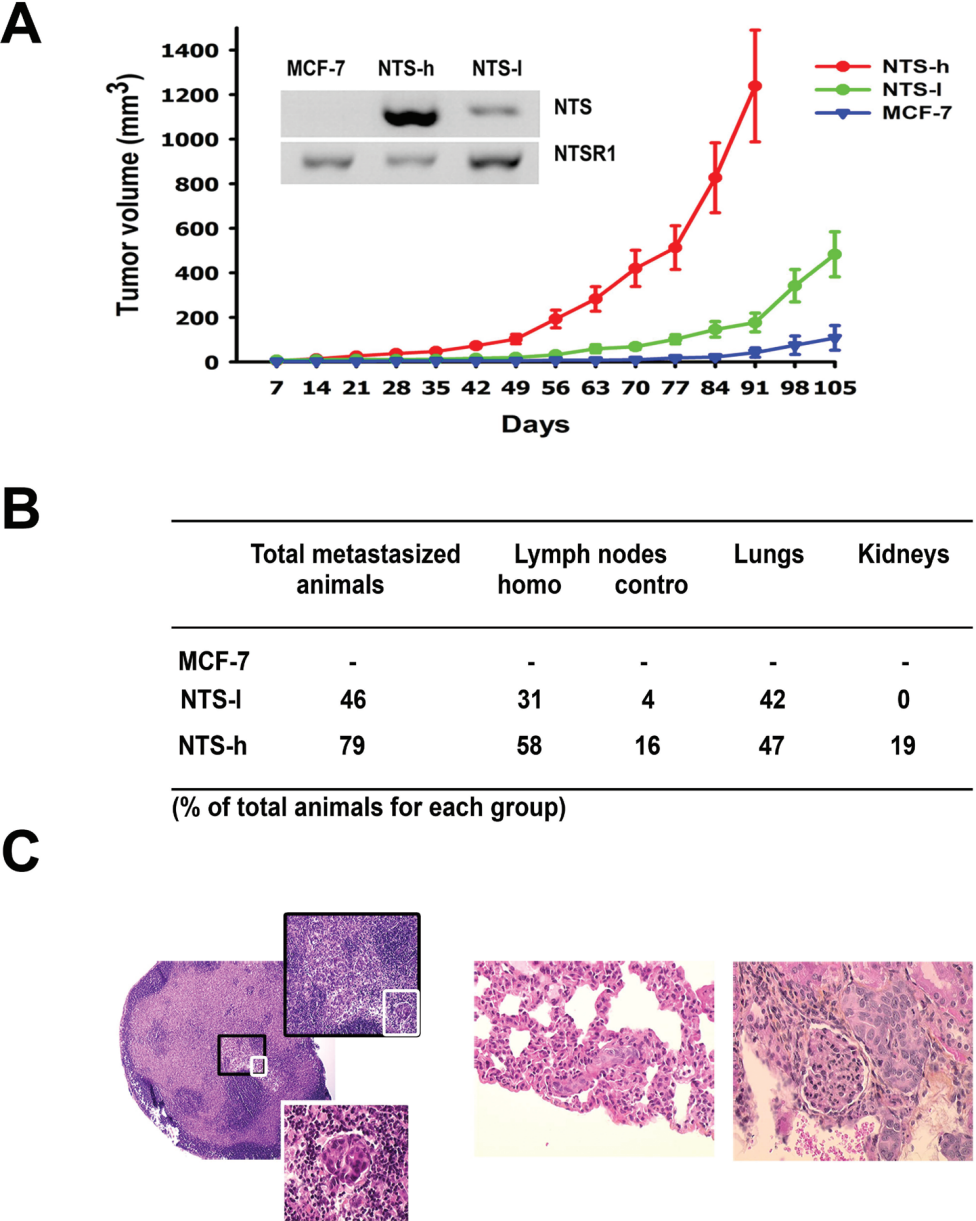
NTS/NTSR1 complex enhanced experimental tumor growth generated in human breast cancer cell lines **(A)** Experimental tumors were generated from the breast cancer cell line, MCF-7 and the NTS-overexpressing subclones. Comparative growth curves of MCF-7, NTS-h (high NTS expression) and NTS-l (low NTS expression) cells xenografted in 10, 20, and 25 mice, respectively. Tumor volumes were measured every week. Inset, NTS and NTSR1 transcript analysis from 200ng of MCF-7, NTS-h, and NTS-l total RNA. **(B)** Proportion of animals with metastases, and metastases distribution within organs and lymph nodes. **(C)** Typical H&E staining performed on paraffin sections of (left) invaded lymph node at 50X magnification, 200X magnification (black square) or 400X magnification (white square); (middle) lung metastasis at 400X magnification; (right) kidney metastasis at 400X magnification.

MCF-7 cells, which constitutively express NTSR1, were transfected with the neurotensin full length coding sequence to evaluate the influence of an autocrine neurotensinergic signaling loop on the tumorigenicity of the ER-positive breast cancer. Amongst the selected clones, two showed a differential NTS expression based on transcription levels the NTS high expressing clone, NTS-h, and the NTS low expressing clone, NTS-l (Figure 1A inset). The NTS expression levels were confirmed by quantitative RT-PCR in both clones, showing a 500-fold and a 4-fold NTS transcript's induction for NTS-h and NTS-l, respectively as compared to the MCF-7 parental cells (Figure 1SA). High expression levels of NTS alter the cell morphology by reducing the size of the cytoplasm. The nucleocytoplasmic ratio is similar for NTS-l and parental cell lines (1/3.2 and 1/3.3 respectively) whereas the ratio decreases to (1/2.5) for NTS-h cells (Figure 1S B). The size of the nucleus remained similar for the three cell lines (Fig 1S B). Immunofluorescent staining experiments show the sub-cellular localization of NTS (Figure 1S C - panels 1, 2, 3), and NTSR1 (Figure 1S C - panels 4, 5, 6). In wild type cells, no or an extremely weak NTS labeling is noted. In the NTS-l cells, NTS immunoreactivity is dispersed in small dots throughout the cytosol. The same pattern is observed in NTS-h cells with larger dots of stronger intensity due to the higher NTS expression level. In parallel, in MCF-7 cells, NTSR1 labeling is localized at the cell membrane, whereas an intense intracellular granular labeling of an endocytosed receptor is seen in NTS-h cell. NTS-l cells show a dual pattern of NTSR1 localized both at the membrane and inside the cytoplasm.

To evaluate the influence of the NTS autocrine signaling loop on tumor growth, the MCF-7 parental line and the two NTS-overexpressing clones, NTS-l and NTS-h, were xenografted in the mammary fat pad of female athymic mice. Tumor growth rose with NTS expression levels (Figure 1A). The group bearing the MCF-7 cells began to develop tumors after 77 days to reach a small size of 108 ± 56 mm3 at day 105. During the same period of time mice bearing NTS-overexpressing cells developed much bigger tumors, with a volume of 483 ± 102 mm3 for NTS-l at day 105 and 1239 ± 251 mm3 for NTS-h at 91 days. The corresponding tumor weights were 0.2 ± 0.1 g, 1.0 ± 0.2 g and 2.0 ± 0.2 g, respectively. None of the animals xenografted with MCF-7 cells developed metastases during the experiment (Figure 1B). On the contrary, metastases were observed in 41% of the NTS-l group and 76% of the NTS-h group. The metastases were preferentially found in the homolateral lymph nodes (24% and 59% respectively) and the lungs (35% and 47% respectively). Furthermore, 18% of the animals in the NTS-h group also showed a metastatic spread in the controlateral lymph nodes reflecting a more advanced metastatic stage. Immunohistochemical slides representing examples of a lymph node, a lung, and a kidney metastatic lesion are shown in figure 1C.

### The NTS/NTSR1 complex enhances EGFR, HER2, and HER3 expression and activation in breast cancer and tumoral cells

**Figure 2:**
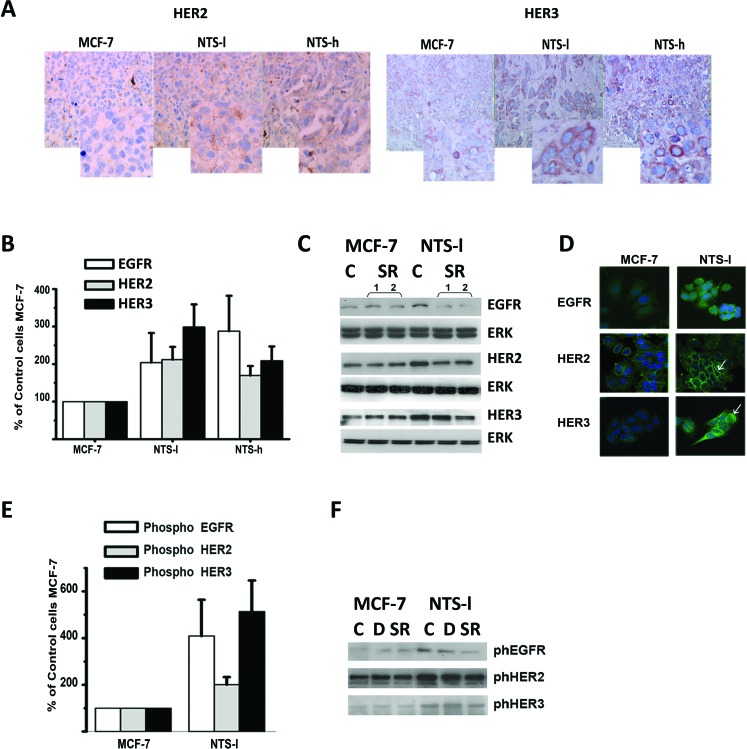
NTS autocrine and paracrine regulation enhanced EGFR, HER2, and HER3 basal expression and activation in human breast cancer cell lines **(A)** HER2 and HER3 immunohistochemistry performed on paraffin embedded tumors from mice xenograph with MCF-7, NTS-l or NTS-h. 200X magnification and computer enlargement of specific areas. **(B)** Breast cancer cells MCF-7, NTS-l and NTS-h, with the histograms representing intensity-based quantification of Western blot bands of basal total protein, EGFR, HER2, and HER3, using Morpho Expert software (Explora Nova, France). Values are expressed as the percentage of the control MCF-7 cells and are the mean ± SEM of 5 to 7 independent experiments. **(C)** Representative western blot analyses of EGFR, HER2, HER3 and ERK 1/2 total protein from MCF-7 and NTS-l cells treated with 5x10^−6^ M SR 48692. **(D)** EGFR, HER2, and HER3 immunolabeling in MCF-7 and NTS-l cells treated after 48h of seeding. **(E)** Breast cancer cells MCF-7 and NTS-l, with the histograms representing intensity-based quantification of Western blot bands of phosphorylated protein, EGFR, HER2, and HER3. Values are expressed as the percentage of the control MCF-7 cells and are the mean ± SEM of 5 to 7 independent experiments. **(F)** Representative western blot analyses of Phospho EGFR, phosphoHER2, and Phospho HER3 protein from MCF-7 and NTS-l cells treated with DMSO or 5x10^−6^ M SR 48692 for 48h.

In order to identify the mechanisms underlying NTS induced growth and metastasis, we searched for the possible interrelation between NTS/NTSR1 complex and epidermal growth factor receptors (HERs). We first observed the amplification of HER2 and HER3 protein levels in tumors obtained after the xenograft of NTS-h and NTS-l cells, as compared to MCF-7 cells. In these cases, the membranes of the labeled tumoral cells was often thicker and more intense (Figure 2 A).

Analysis of NTS-l, NTS-h, and MCF-7 cellular protein content, showed an increase of 150–275% of EGFR, HER2, and HER3 (Figures 2B and C). This effect was confirmed by the stronger labeling of all three HER receptors by immunocytochemistry in NTS-l cells as compared to MCF-7 cells (Figure 2D). In NTS-l cells, EGFR is accumulated in clusters close to the nucleus, while in MCF-7, EGFR is at the membrane, HER2 membrane labeling, and HER3 cytosol labeling are more intense in NTS-l than in MCF-7 cells (Figure 2D see white arrow). The specific NTSR1 antagonist, SR 48692, reduced HERs increases in NTS expressing cells, but not in wild type cells (Figures 2C and 2S A), validating the contribution of NTS/NTSR1 complex in this HERs overexpression. As HERs transcript levels were essentially the same in MCF-7 and NTS-l cells (Figure 2S B), HERs protein accumulation in the cells suggest that recycling and degradation are altered due to NTS exposure.

In parallel, an increase in the activation states for all three receptors was observed in NTS-l cells. The phosphorylation levels were enhanced up to 400 and 500% for EGFR and HER3, respectively (Figures 2E and F), and was partially abolished by the antagonist SR 48692 (Figure 2S C). Similar observations were made with NTS-h cells and a 200% increase was seen for the three receptors (not shown).

**Figure 3:**
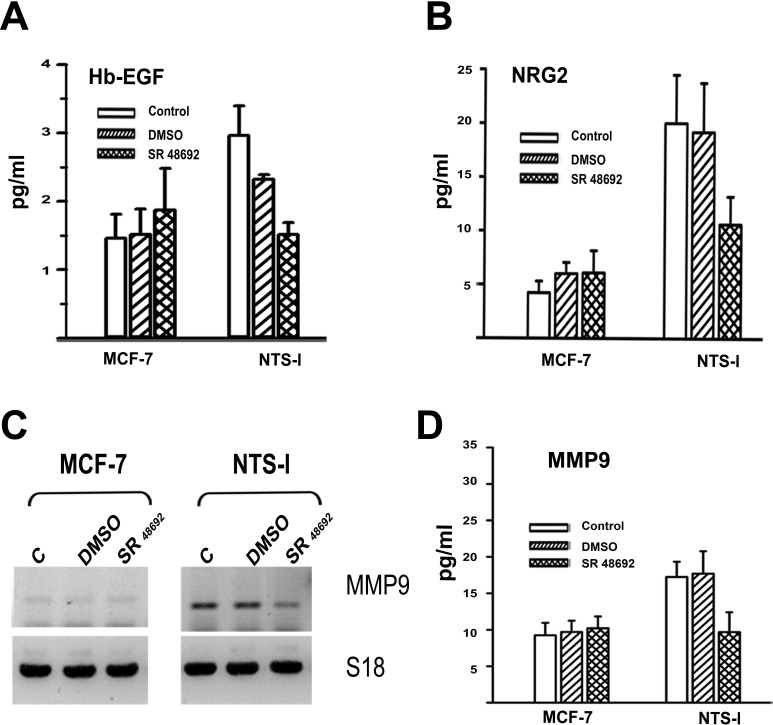
EGF like ligands and metalloprotease released by NTS **(A)** Amount of Hb-EGF (pg/ml), assayed in 0% FCS culture media of MCF-7, or NTS- l, cells. Cells were not treated, or treated for 24h with DMSO, 5x10-6M, SR 48692. Using Paired t test p = 0.0088 between DMSO and SR 48692 NTS-l treated cells, n=4; with unpaired test p=0.03 between MCF-7 and NTS-l, n=5. **(B)** Amount of NGR2 (pg/ml) assay in 0% FCS culture media of MCF-7 or NTS-l cells not treated or treated for 48 h with DMSO, 5x10-6M SR 48692. Using Paired t test p = 0.005 between DMSO and SR 48692 NTS-l treated cells, n=5; with unpaired test p=0.016 between MCF-7 and NTS-l, n=4. **(C)** MMP9 transcript analysis of total RNA from MCF-7, and NTS-l treated with DMSO or 5x10^−6^ M SR 48692 for 48h. **(D)** Amount of MMP9 (pg/ml) assay in 0% FCS culture media of MCF-7 or NTS-l cells not treated or treated for 48 h with DMSO, 5x10^−6^M SR 48692. Using Paired t test p = 0.005 between DMSO and SR 48692 NTS-l treated cells, n=5; with unpaired test p=0.003 between MCF-7 and NTS-l, n=5.

Subsequently, we searched for a possible EGFR and HER3 autocrine activation by the release of EGF “like” ligands from the cell membrane following a proteolysis process mediated by NTS. In the culture media from NTS-l cells, the amount of HB-EGF was twofold higher than MCF-7 cells. The presence of SR 48692 in the culture media abolished this increase (Figure 3 A). In the same vein, a stronger release of neuregulin 2 could be detected in the culture media of NTS-l cells compared to MCF-7 cells, and again counteracted by SR 48692 (Figure 3 B). The release of these specific ligands for EGFR and HER3 suggests an activation of metalloproteases due to NTS exposure. MMP9 transcripts increased (Figure 3 C) in NTS-l cells as compared to MCF-7, as was the case for MDA-MB 231 cells [16]. In addition, MMP9 anchored at the membrane was activated, as a 180% increase of MMP9 released in culture media was detected in NTS-l as compared to MCF-7 cells and abolished by SR 48692 (Figure 3 D).

**Figure 4:**
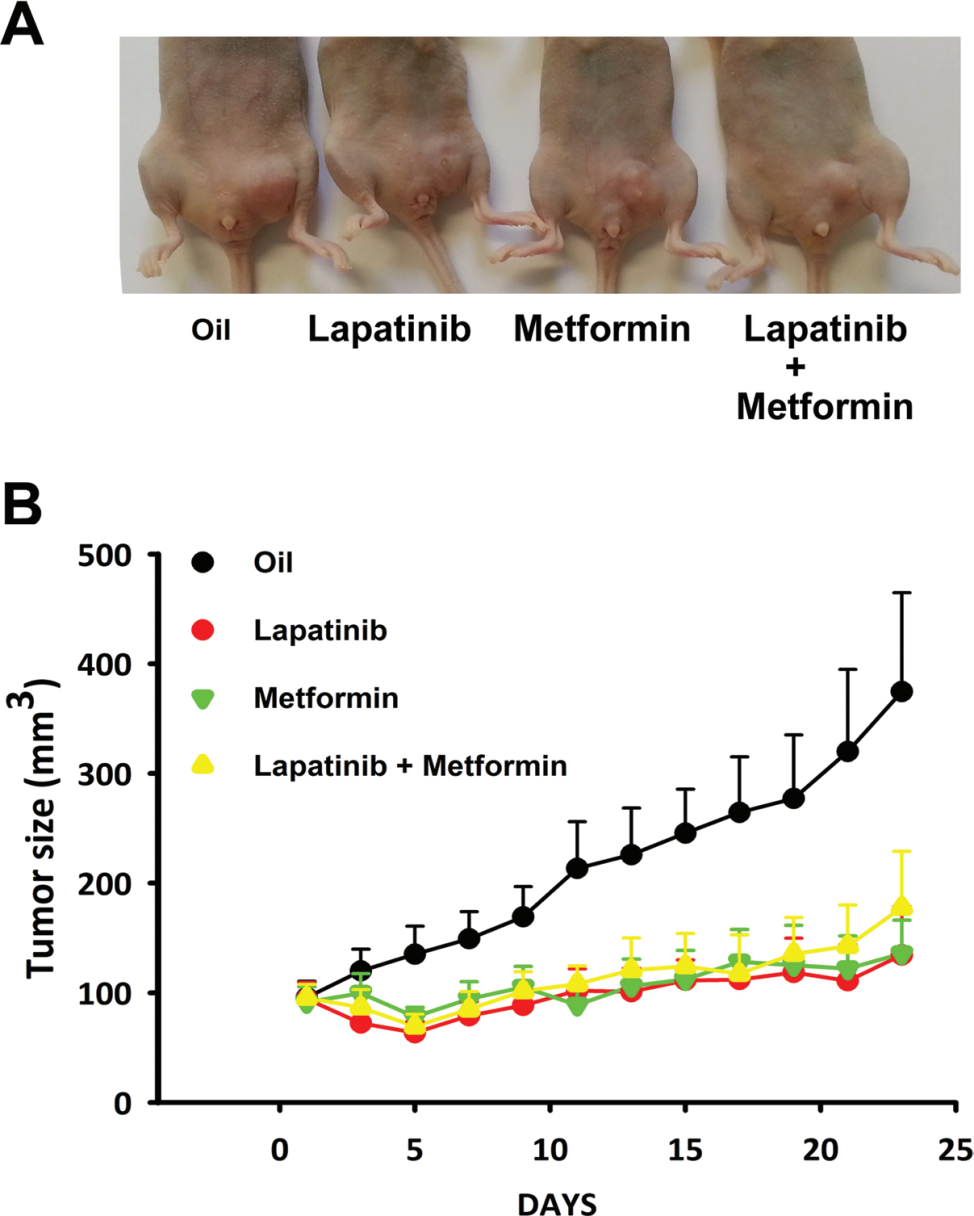
NTS/NTSR1 expressing tumors response to EGFR/HER2 inhibitor treatments **(A)** NTS-h cells were inoculated in the left mammary gland of the mice. Here is shown an example of a mouse from each group after 23 days of treatment. **(B)** Tumor growths generated by NTS-h cells treated for 23 days with sesame oil 6% DMSO, or 75 mg/kg lapatinib, or 200 mg/kg metformin, or both. At day one, 7 mice per group were randomized on tumors size reaching approximately 95 mm^3^.

We confirmed the contribution of EGFR and HER2 activation in NTS induced tumor growth by treating xenografted mice with NTS-h and lapatinib. Lapatinib inhibits the tyrosine kinase activity of both HER2 and EGFR. This property is currently used in breast cancer treatment. We confirmed that NTS-h cells do not carry the following EGFR active mutations: exon 19 deletion, exon 20 insertion, exon 18 Q719A, Q719C, Q719S and exon 21 L858R, L861Q point mutations. These cells do not possesses the HER2 exon 20 insertion, or the KRAS point mutations: G12D, G12S, G12C, G12R, G12A, G12V, G13D KRAS. Figures 4 A and B show that the tumor growth rate is reduced when mice are treated with lapatinib. The final tumor volume after 23 days of treatment was 352.3 ± 87 mm3 for the control, and 134.5 ± 44.15 mm3 (p= 0.02 vs control) for lapatinib. We also tested metformin on our model. Metformin is suspected to provide anticancer effects in breast cancer and several clinical trials are currently under way [24]. In parallel, metformin was shown to disrupt the crosstalk between insulin receptor and NTS receptor in pancreatic cancer cells [25]. In addition to inhibiting the mTOR pathway, metformin prevents ERK activation induced by NTS and insulin [26]. In a breast cancer cellular model with NTS overexpression, metformin reduced the tumor growth with the same efficiency as lapatinib. No additional effects were detected when both drugs were employed. The final volumes were 135.9 ± 30.4 mm3 and 177.7 ± 51.3 mm3 when animals were treated with metformin or both drugs, respectively (Fig 4 A and B). The tumor doubling time was in agreement with tumor volume with 11.7 ± 1.35 days, for controls. For lapatinib, metformin, and metformin + lapatinib double time cannot be calculated for because within the group 2 to 3 tumors shrank, the others grow very slowly. The absence of additional response suggested a common signaling cascade was targeted by both drugs.

### The NTS/NTSR1 complex enhances pro-metastatic cellular effects

**Figure 5:**
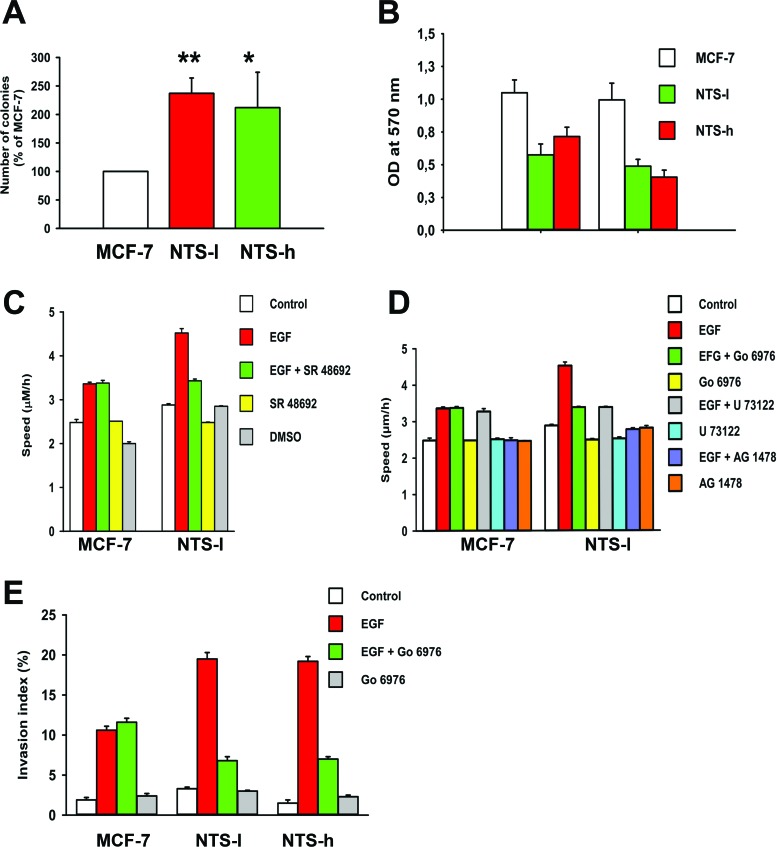
NTS autocrine and paracrine regulations enhanced oncogenic cellular effects on EGF-induced migration and invasion **(A)** Number of colonies formed on semi-solid medium after 12 days expressed as the percentage of MCF-7 cells. **(B)** Adhesion assays were performed on type I collagen supports. After 1h or 48h of seeding, cells were gently washed and the remaining attached cells were quantified by spectrophotometric analysis of crystal violet staining. Results represent the mean optic density ± SEM of 4 experiments. **(C)** Speed of migration on type I collagen of MCF-7 and NTS-l cells, control or treated with EGF (10 ng/mL), in the presence or not of SR 48692 (5x10-6 M). Results represent the mean ± SEM of 9 to 10 independent experiments. **(D)** Speed of migration on type I collagen of MCF-7 and NTS-l cells control or treated with EGF (10 ng/mL), in the presence or not of the PKC-inhibitor Gö6976 (5x10-8M) or the PLC-inhibitor U73122 (5x10-6 M) Results represent the mean ± SEM of 4 independent experiments. Results represent the mean ± SEM of 3 to 4 experiments. Student-Newman-Keuls Multiple Comparisons Test was performed on the data : ***P<0.001,**P<0.01, and *P<0.05. **(E)** Synergism between NTS and EGF on invasion in a type I collagen invasion assay. Cells were seeded on the top of a type I collagen gel and treated with EGF (100 ng/mL) in the presence or absence of Gö6976 (5x10-8 M). Results represent the mean ± SEM of 3 to 4 experiments.

We evaluated the oncogenic cellular effects impacted by NTS. The basal growth capacity of MCF-7 in an anchorage-independent context was doubled in the presence of NTS. In MCF-7 (Figure 5A), the colonies were organized in a spherical conformation around an inside lumen delimited by a single cell monolayer in wild type cells whereas, NTS-h or NTS-l the colonies were larger and the cells forming compact spheroids, filled up with cells (Figure 3S A).

Cell detachment is a pre-requisite in metastatic spreading. For this purpose, we studied the adhesion ability of our cellular models on various matrices. Cells were initially detached from their support and the kinetics of reattachment was evaluated. After 1 hour of seeding, the NTS-h cells displayed only 68% and 60% of the adhesion capacity on collagen (Figure 5 B) and matrigel (Figure 3S B) respectively, as compared to MCF-7. NTS-l cells displayed 55% and 52%, respectively. Interestingly, this decline in cellular adhesion ability stabilizes over time. At 48 hours of post seeding, the percentage of adherent cells is similar to those after 1 hour (Figure 5 B and 3S B). This suggests that NTS alters the basal cell adhesion capacities of the tumor cells.

We performed migration assays on type I collagen or matrigel supports, because migration requires cell interactions with the extracellular matrix. We developed an experimental procedure, in order to estimate the migration speed of the cells. As described in detail in the methods section, this procedure allows measuring the average distance covered by the cells within 48h, while preserving the matrix coating on the dishes. As shown in figure 5 C and 3S C, NTS expression significantly affects the basal migration speed of the NTS-l cells on both collagen and matrigel coated dishes. The migration speed on collagen was 2.50 ± 0.07 μm/h and 3.10 ± 0.09 μm/h (p < 0.0001; n=10) and on matrigel was 3.99 ± 0.21 μm/h and 5.31 ± 0.24 μm/h (p = 0.003; n=12) for MCF-7 and NTS-l, respectively. As EGFR expression was enhanced under NTS autocrine regulation, we inquired on the effect of EGF on cell speed migration. When cells were treated with EGF, a clear synergic effect between NTS and EGF was observed. The NTS-l cells migrate 38% and 42% faster when treated with EGF on collagen and matrigel respectively, compared to EGF-treated MCF-7 cells (Figure 5 C and 3S C). In order to confirm the contribution of the NTS-NTSR1 complex in this synergic effect, MCF-7 and NTS-l were treated with EGF and the NTSR1 specific antagonist SR 48692. As shown in figure 5 C and 3S C, this treatment had no effect on the EGF-induced migration of MCF-7 on collagen and matrigel, whereas, SR 48692 inhibited the acceleration of the migration speed in the EGF-treated NTS-l cells. Specific PKC and PLC inhibitors were applied and clearly abolished the synergic effect of NTS and EGF observed in NTS-l cells. In the parental cells, EGF's contribution to the migratory effect was insensitive to these inhibitors although the basal migration speed did increase in matrigel matrix (Figures 5 D and 3S D). As control, a specific inhibitor of EGFR, the AG1478, completely abolished the EGF-induced migration in both MCF-7 and NTS-overexpressing cells (Figure 5 D and 3S D).

The invasiveness properties of NTS-overexpressing cells, was studied using a 3 dimensional collagen invasion assay. Results are expressed as the invasion index corresponding to the number of invading cells related to the number of total seeded cells. The introduction of NTS expression into MCF-7 cells induced a small increase in invasiveness properties (Figure 5 E). However, EGF-induced invasion doubled in NTS-overexpressing cells (20% invasion index) as compared to MCF-7 (10% invasion index). The induction of invasiveness was inhibited by PKC inhibitors only in NTS-overexpressing clones, suggesting dependence of this effect on GPCR activation.

**Figure 6:**
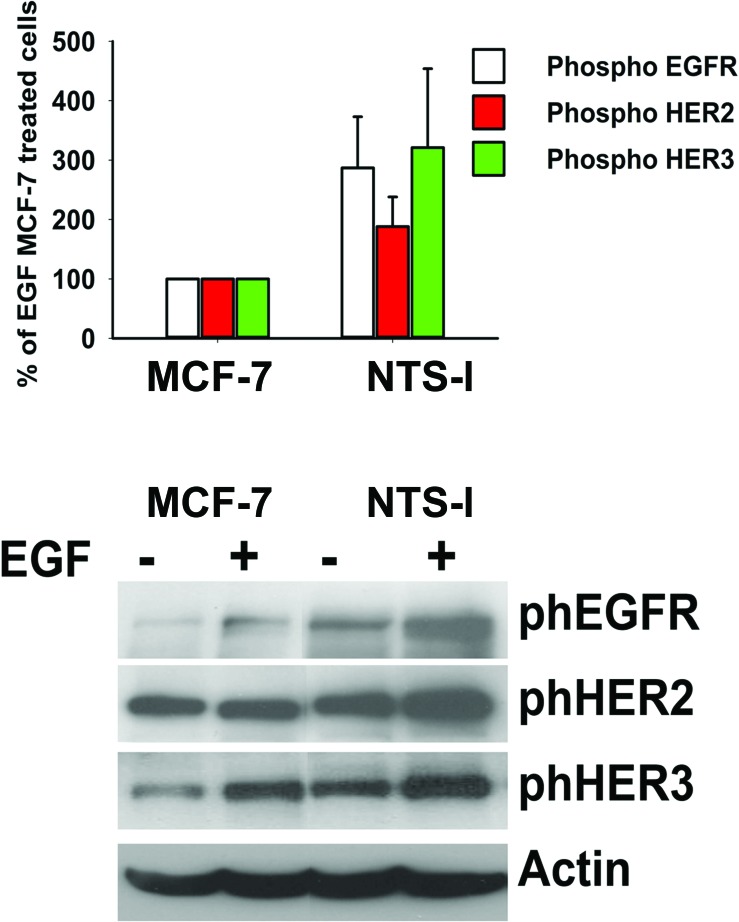
Synergy between NTS and EGF to activate EGFR, HER2, and HER3 **(A)** Breast cancer cells NTS-l or MCF-7, with the histograms representing intensity- based quantification of Western blot bands of phosphorylated protein, EGFR, HER2, and HER3 treated for 10 min with 10ng/ml EGF. Values are expressed as the percentage of the EGF treated MCF-7 cells and are the mean ± SEM of 5 independent experiments. **(B)** Representative Western blot analyses of phosphoEGFR, phosphoHER2, phosphoHER3 and actin from MCF-7 and NTS-l cells treated or not with 10ng/ml EFG for 10 min.

The synergic effects of NTS and EGF on cellular migration and invasion suggest that a new pattern of HER heterodimers, with a higher EGF response potency, is generated in the cells. When cells were stimulated with EGF, the amount of phosphorylated proteins EGFR, HER2, and HER3 in the NTS-overexpressing cells is higher than the phosphorylated proteins in wild type cells, with an increase of 290, 190, and 275%, respectively as compared to MCF-7 EGF treated cells (Figure 6 A and B).

### Overexpression of NTS and NTSR1 correlates with HER2 and HER3 overexpression in breast human tumors

**Figure 7:**
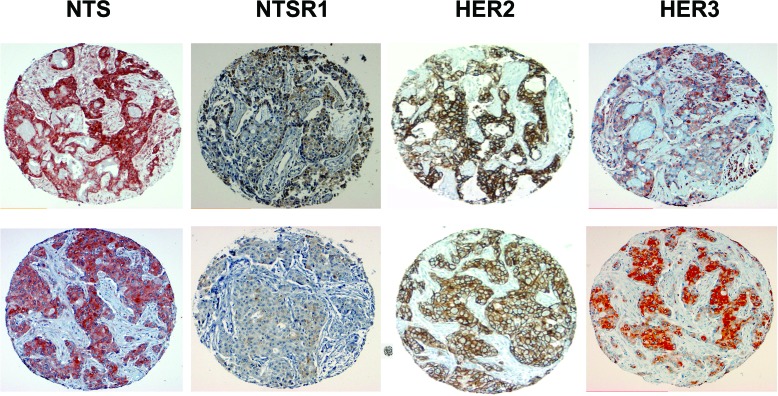
NTS, NTSR1, HER2, and HER3 immunohistochemistry on breast and lung cancer tumors Two examples of positive labeling scored 2 or 3 for NTS, NTSR1, HER2, HER3 from breast tumor TMA, labeling was performed on consecutive slides. 100X magnification.

Co expression of NTS, NTSR1, HER2 and HER3 was analyzed in breast cancer tissue microarrays (TMA). Seven TMAs containing 269 samples each, was scored from 0 to 3 according to the labeling intensity and the proportion of stained cells. Correlations between the expression of NTS/NTSR1 complex and the expression of HER2 and/or HER3 were evaluated. We considered that a score of 2 and 3 indicate the overexpressed condition, and consequently we performed contingent's analysis on these combined scores. Results are summarized in table 1. NTS and NTSR1 were found in 23% of the samples, in agreement with our previous studies [14], whereas HER2 and HER3 were found in 28% and 53% of the samples, respectively. Amongst this NTS-NTSR1 positive population a higher proportion overexpressed HER3 (16%) than HER2 (7%). HER3 expression was positively associated with those for NTS and NTSR1 (OR= 19.073, 95% Confidence Interval [13.633-26.684], p<0.0001). In contrast, no significant relation between HER2 and NTS/NTSR1 was found. Only 5% of the samples overexpressed the four markers, nevertheless HER2 and HER3 expressions remained strongly significantly associated to NTS/NTR1 expression (OR = 12.117 95% Confidence Interval: to [7.121-20.618], p<0.0001). It should be noted that an absence of correlation between high expression of HER2 and HER3 was detected. Two examples of the same tumor sample labeled for the four markers are shown in figure 7.

**Table 1:** Contingent analysis for NTS, NTSR1, HER2 and HER3 expression on samples from breast cancer tissue microarray

	NTS	NTSR1	HER2	HER3
n total	1408	1347	1268	1410
Positive n (%)	1029 (73)	375 (28)	361 (28)	753 (53)
NTS, n (%)		301 (23) *p*<0.0001	274 (22)	599 (43) *p* = 0.006
NTSR1, n (%)			102 (8)	244 (19) *p*<0.0001
HER2, n (%)				218 (18)
NTS & NTSR1, n (%)			85 (7)	204 (16) *p*<0.0001
NTS & NTSR1 & HER2, n (%)				62 (5) *p*<0.0001

n total= number of sample readable; Positive = number of sample scored 2 or 3; P = Fisher's Exact Test

## DISCUSSION

The organization of tumor cell signaling networks evolves with accumulation of genetic and epigenetic alterations in connection with the local stroma, vessels and immune system. Identifying factors which contribute and predict tumor aggressiveness are means to understand how the progression of the disease evolves across individuals. Unfortunately, the biological and clinical validation of these factors is difficult because tumors are often heterogeneous and their molecular characteristics evolve over time. In addition to the clinical parameters, genetic alterations on key genes provide additional information on disease outcome. These genetic alterations can be modulated by regulatory mechanism which may cause changes to the amplitude of cell aggressiveness within the tumor. While genetic alterations acquired by tumors are irreversible, their regulatory process can be de-programmed to restore a tumor phenotype to a less aggressive and more differentiated form.

In this article we show that the NTS/NTSR1 complex enhances tumor aggressiveness (tumor growth and metastasis emergence) by enhancing HERs expression, and their activation by the release of specific EGFR and HER3 ligands. This concept can be extended to other tumors, as we observed similar results in lung cancer cells and subsequent experimental tumors [27]. In lung cancer cells all three receptors (but mainly HER2 and HER3) are over expressed under NTS autocrine regulation. EGFR and HER3 activation occurred with HB-EGF and NGR1 as well as MMP1 released from the cells [51].

In breast cancer cells, NTSR1 activation alters many cellular effects having oncogenic characteristics including proliferation, survival, adherence, migration and invasion, with a synergic effect between NTS and EGF on cellular migration and invasiveness. This latter point may explain the exacerbation of the metastasis process seen due to NTSR1 activation. Synergy between NTS and EGF was previously described on DNA synthesis in primary adult rats' hepatocytes [28], and the regulation between these two factors appears to be independent of oncogenic characteristics of the cells. EGF was detected in normal and tumoral breast tissues, with a higher expression found in normal tissues adjacent to tumor [29]. We previously showed that NTS is expressed in normal breast epithelial cells. Its expression is regulated by estrogen [14]. The synergy between NTS and EGF may therefore occur during the breast carcinogenesis.

Our data showed that under NTS stimulation, EGFR, HER2 and HER3 are up-regulated and intrinsically activated. This over expression was not associated with gene transcriptional or post-transcriptional activity, suggesting that NTS induced a new equilibrium in HERs cellular traffic and a new pattern of HERs formed after stimulation. HERs internalization pathways depend on the expression of ligands and/or their receptors [30,31]. Under physiological conditions (low concentration of ligands and moderate EGFR expression <200 000/cell), EGFR internalization is dependent on clathrin coated pits, with a half-life of 6-10 hours. However, under conditions of receptor overexpression or high concentration of ligands, the clathrin pathway becomes saturated, and the complex (ligand-receptor) is internalized with a slow kinetics in a clathrin independent pathway. Under these conditions, the half-life can reach 24 hours [32,33].

In cells overexpressing HER2, receptors are mainly localized at the cell membrane, indicating that overexpression of HER2 does not lead to accelerating its endocytosis [34].The HER2 heterodimerization with EGFR influences the endocytosis pathway for both receptors. Treatment by EGF of cells expressing low HER2, resulted in HER2 down-regulation [35]. In contrast, EGFR activation in cells overexpressing HER2, does not affect the membrane expression of HER2 or its degradation [34,36]. In addition, overexpression of HER2 had a dominant-negative effect on the down regulation of stimulated EGFR, and stabilized both receptors by altering autophosphorylation patterns [34,37]. HER2 overexpression may also prevent EGFR internalization by clathrin-dependent endocytosis, and it's re-directed from the degradation to the recycling pathway [36,38,39].

The activation of HER3 leads to its internalization and its down regulation. However, HER3 internalization is slower than EGFR internalization [40]. In addition, HER3 is inefficiently sorted to the degradation pathway, apparently due to the lack of the C-terminal domain, which contains patterns used for targeting to lysosomes [41,42]. It has also been suggested that neuregulins do not guide HER3 to degradation due to the early dissociation of complexes (ligand-receptor) in endosomes [41]. In these situations the stabilization of HERs cells are independent of gene transcription, activation and amplification, as in the case for NTS.

Associated with HERs regulation, we also observed an increase of NRG2 and HB-EGF secretion by NTS mediated by MMP activation. The release of these growth factors caused the concomitant activation of HER3 and EGFR. EGF “like” ligands are largely implicated in breast cancer progression, yet, most reports have only studied the expression of ligands specific to EGFR, or those specific to HER3. For example, in one case a 10 fold increase of HB-EGF in cancer tissues was reported, and in another a high proportion of the four neuregulins and major isoforms were expressed in the cytoplasm of DCIS and IDC of the breast [29,43,44]. In both cases, deleterious effects of growth factors are often in HER2 overexpressing cells, indicating that EGFR/HER2 and/or HER3/HER2 dimers are related to biological aggressiveness [29]. Globally, the overexpression of both HER2 and HER3 participates in the stabilization of the HERs dimer, and subsequently the sustained activation of the HERs, and the survival pathway. By enhancing the overexpression and activation of EGFR, HER2 and HER3, the NTS/NTSR1 complex becomes an upstream factor that modulates this regulatory mechanism.

In agreement with *in vitro* studies, tumor growth induced by NTS/NTSR1 can be restrained by a specific tyrosine kinase inhibitor EGFR and HER2, lapatinib. The cascade of biological events from the interaction between NTS and NTSR1 to the activation of HERs receptor appears to be a major contributor to rapid cancer cell growth.

This cascade is inhibited by metformin, known to interfere with activated mTOR and ERK pathway [26,45]. The lack of additional effects from the combination of the two drugs suggests that consecutive cellular events lead to increase tumor aggressiveness by NTS. We previously showed that sustained NTSR1 activation generated a permanent PKC dependent activation of ERK signaling [46]. It is therefore coherent that metformin and other ERK or PKC inhibitors counteract the same NTS oncogenic cellular effect. PKC appears to be a central signaling hub to generate cell aggressiveness by NTS/NTSR1 through the sustained overexpression and activation of HERs.

Experimental tumors report the tumorigenic performance of single cell lines or clones. Nevertheless, heterogeneity is an important characteristic of human tumors. For instance, we found that in 35% of patient with IDC, NTSR1 was expressed in at least 80% of tumoral cells, and only 20% of patients express high level of NTSR1 and NTS [47]. In human tumors, NTS and NTSR1 are expressed in cells clusters with diverse sizes. Tumoral cells with potential aggressiveness characteristics could be detected with NTS/NTSR1 labeling, and specific treatment could be proposed accordingly. Lapatinib is proposed in a second line of treatments, in combination with other drug in advanced or metastatic breast cancers whose tumors overexpress HER2. Our results suggest that a more restrained (or targeted) population, can be determined by taking into account NTS and NTSR1 co expression. The resulting subpopulation will provide a significantly better performance for this drug.

## CONCLUSION

The activation of the neurotensinergic system in breast tumors induces the overexpression of the EGFR, HER2 and HER3 receptors and their concomitant autocrine activation. The presence of this regulatory mechanism would have a significant impact on cancer progression in tumor cells by accelerating the process of metastasis. It also modulates the response to tyrosine inhibitor HER2 and EGFR therapy. NTS/NSTR1 overexpression in breast cancer cells creates a tumoral context for EGFR tyrosine kinase inhibitor responsiveness, and therefore a new population of patients would be eligible to these specific tyrosine inhibitors.

## MATERIALS AND METHODS

### Cell culture procedure

The human breast adenocarcinoma cell line MCF-7 and the corresponding NTS–overexpressing clones, NTS-h (high level of NTS) and NTS-l (low level of NTS), were grown at 37°C, in a humidified atmosphere of 5% CO2, in DMEM supplemented with 10% fetal calf serum, 2 mM glutamine and G418 0.5 mg/mL (Invitrogen™).

### Tumor xenografts

3 millions cells (MCF-7, NTS-h, NTS-l) resuspended in Matrigel (BD Biosciences) were then inoculated in the right mammary gland of the mice. Tumor growth was induced by a daily intra-peritoneal injection of 2μg estradiol per mouse. Tumors, axillary and inguinal lymph nodes, lungs, liver and bones (vertebra, sternum) were taken, weighed and fixed in formol or frozen. All the procedures were in accordance with the “Guide of the Care and Use of laboratory Animals”. Institutional Review Board approval was obtained by «Le Comité d'Ethique pour l'Expérimentation Animale Charles Darwin # Ce5/2010/049 ». For lapatinib experiments NTS-h cells were inoculated in the left mammary gland of the mice. 51 days after injection, 4 groups of 7 mice were randomized as follows : 95.9 ± 14.57 mm3 for control group, 94.5 ± 15.0 mm3 for lapatinib group, 91.5 ± 14.09 mm3 for metformin group and 95.6 ± 12.20 mm3 for lapatinib and metformin group. Mice were treated for 21 days per os, with sesame oil containing 6% DMSO, or 75 mg/kg lapatinib, or 200 mg/kg metformin or both.

### Western blots

Samples in Laemmli buffer were loaded on 10% SDS-PAGE and transferred to PVDF membranes. Membranes were exposed to primary antibody overnight. Total anti-EGFR (1:500), anti-phospho-EGFR (1:500), anti-phospho-HER2 (1:500), anti-HER3 (1:2000), anti-phospho-HER3 (1:1000), anti-ERK 1/2 (1:2000) were from Cell Signaling Technology®. Total anti-HER2 (1:2000) were purchased from Neomarkers and anti-βactin (1:50000) from Sigma®. Secondary anti-rabbit (Santa Cruz Biotechnology) or anti-mouse (Sigma®) antibodies conjugated to HRP were used at 1:2000 dilutions for 1h at room temperature and visualized by enhanced chemiluminescence (GE Healthcare®). See details in supplementary information.

### Adhesion assays

The assay was performed in 96-wells plates coated or not with 50 μg/mL of type I collagen (Sigma®) overnight at 4°C or with 1:10 diluted-growth factor reduced Matrigel (BD Biosciences®) for 1 hour at 37°C. Cells were harvested with PBS containing 0.5 mM EDTA for 20 min at 37°C, pelleted and suspended in adhesion buffer (DMEM, 15 mM HEPES, 1.2g/L sodium bicarbonate, 0.2% BSA). 5x10^4^ cells per well were seeded and incubated at 37°C for 1 or 48 hours. Wells were washed with culture medium and adherent cells fixed with 5% paraformaldehyde for 45 min, then colored with 0.1% cristal violet during 30 min at room temperature. Cells were subjected to 30 min lysis in 1% SDS under agitation. Absorbance at 570 nm was determined by spectrophotometric measures and correlates with the number of adherent remaining cells.

### Anchorage-independent growth assay in soft agar

Colony growth assays were performed by seeding on the top of a 0.6% low gelling temperature agarose layer, 5x103 cells in 0.5 mL of cultured medium containing 0.3% agarose solution ± EGF (10 ng/mL). Culture medium and EGF were replenished every 2-3 days. Cells were incubated at 37°C for 12 days and colonies ≥ 50 μm were counted in the whole well.

### Cell migration assays

12 wells-culture plates were coated with type I collagen or Matrigel as mentioned in adhesion assay. 1x105 cells were plated for 4 hours in an 8 mm-cloning ring placed in the center of the well to form a confluent circle of monolayer cells. The cylinder was removed and cells grow for 16 hours. Cells were then treated in serum-free medium ± EGF (10 ng/mL) in the presence or absence of various cell signaling inhibitors. Four pictures were acquired per well at the initial time of treatment and 48 hours later for comparison with an inverted microscope at 200X magnification. Migration speed was determined using the Morpho Expert software (Explora Nova) and was expressed as the average cells covered distance divided by the experimental duration (μm/h). Presented results are expressed in percentage of the non-treated condition.

### Collagen invasion assays

Invasion potential was evaluated by a single-cell collagen invasion model, extensively described [48]. Briefly, 6-wells plates were coated with 1.25 mL of a 1 mg/mL collagen type I solution, allowed to gel for at least 1 hour at 37°C in a humidified atmosphere containing 10% of CO2. 2x10^5^ viable single-cells, obtained by mild enzymatic dissociation with trypsin/EDTA solution and filtration, are seeded on the top of the gel in presence or not of EGF (100 ng/mL) and/or Gö6976 (4x10^−8^ M) for 24h. Invasion index (cells with invasive extensions versus total number of cells × 100) was determined by counting the number of invading and non-invading cells present in 10 to 15 random fields of an inverted phase-contrast microscope.

### ELISA (Enzyme-linked immunosorbent assay)

Cells were seeded at 4X10^6^ cells in 100 mm dish in culture media. The next day media was changed to 4.5 mL of serum free media for 48h. To assay Hb-EGF and NRG-2, the media were concentrated with dialysis tube (7 Spectra / Por ® Dialysis Membrane). In all samples Protease Inhibitor Cocktail P8340 [1:100] were added. MMP9, HB-EGF and NRG2 released in the culture media were assayed by ELISA kits (DuoSet®ELISA Development System, and USCN Life Science Inc.).

### RNA extraction and RT-PCR

Total RNA was extracted by the acidic phenol/chloroform guanidine thiocyanate method as documented by [49,50]. Detailed are described in supplementary information.

### Immunohistochemistry

Procedure is detailed in supplementary information. The slides were incubated at 4°C overnight with primary antibody included anti-NTS (1:200, SC-20806, Santa Cruz biotechnology®), and anti-ErbB3 (1:50, NCL-c-erbB-3, Novocastra™), anti-NTSR1 (1:100; SC-7596, Santa Cruz Biotechnology®) and anti-ErbB2 (1:400, A0485, Dako) was incubated at room temperature for 1hour and 30 minutes respectively. The levels of staining were scored based on staining intensity within the tumor sample, with weak as 1, moderate as 2, strong as 3.

### Patients

We studied 499 specimen from patients operated for breast cancer in 2008 in Algeria. The average patient age was 51.1 ± 11.7 years. The sizes of the tumors were 36.3 ± 21.7 mm. The SBR histoprognostic grading was 6% grade I, 63% grade II and 31% grade III (See supplementary information). Investigation has been conducted in accordance with the ethical standards and according to the Declaration of Helsinki and according to Algerian guidelines and has been approved by the authors' institutional review board from CPMC. Data were analyzed anonymously.

### Statistics

Statistical analysis was carried out using test Student's t-test or Student-Newman-Keuls Multiple Comparisons Test : ***p<0.001,**p<0.01, and *p<0.05. For contingency analysis Fisher's Exact Test was applied. The Odds ratio was performed using the approximation of Woolf.

### ADDITIONAL METHODS

### Transfection procedure

Following the manufacturer's instructions, a pCDNA3.1 plasmid encoding the large fragment of rat neurotensin ou neurotensin full length coding sequence was transfected in MCF-7 cells using the Lipofectamine Plus Reagent (Invitrogen™). Briefly, 0.8x10^6^ cells were plated in 60 mm-Petri dishes and were allowed to grow 24 hours before transfection. 2μg of DNA were complexed together with the Plus Reagent and the Lipofectamine Reagent to form transfection complexes which were incubated during 4 hours on the cells. Complete medium was then added to reach a 10% final fetal calf serum concentration. 24 hours later, cells were harvested and placed in fresh culture medium. Selection was performed 2 days after transfection with 1mg/mL of G418 (Invitrogen™). Stable clones were obtained by cloning dilution and screened by classic RT-PCR and real-time PCR for the NTS expression level.

### Western blot

2x10^6^ cells were grown for 72h then serum-starved for 48h in a phenol red-free medium in presence or absence of different concentrations of 5x10^−6^ M SR 48692 and 25x10^−9^ M MMP9 inhibitor (Calbiochem®). For EGF treatments, cells were then treated 15 min with EGF (10 ng/mL) lysed (20 mM Tris pH 8.0, 150 mM NaCl, 5 mM MgCl_2_, 0,5% NP40, 0,5% glycerol, 1 mM PMSF, protease and phosphatase inhibitor cocktail) at 4°C for 30 min. Samples in Laemmli buffer were loaded on 10% SDS-PAGE and transferred to PVDF membranes. Saturation was performed 30 min at room temperature in 5% non-fat dry milk in TBS 0.1% Tween 20. Primary antibodies were incubated overnight at 4°C according to the manufacturer's instructions. Total anti-EGFR (1:500), anti-phospho-EGFR (1:500), anti-phospho-HER2 (1:500), anti-HER3 (1:2000), anti-phospho-HER3 (1:1000), anti-ERK 1/2 (1:2000) were from Cell Signaling Technology. Total anti-HER2 (1:2000) were purchase from Neomarkers and anti-βactin (1:50000) from Sigma. Secondary anti-rabbit (Santa Cruz Biotechnology®) or anti-mouse (Sigma®) antibodies conjugated to HRP were used at 1:2000 dilutions for 1h at room temperature and visualised by enhanced chemiluminescence (GE Healthcare®).

### RNA extraction

Total RNA was extracted by the acidic phenol/chloroform guanidine thiocyanate. Two cycles of phenol-chloroform extraction (pH 4.7) were preceded by two ethanol precipitations in GTC buffer (4 M Guanidium Thiocyanate, 50 mM Tris pH 7.5, 10 mM EDTA pH 8.0, 30% N-lauroylsarcosine sodium salt, 1% β-mercaptoethanol) and followed by a final extraction with chloroform-isoamyl alcohol 25:1 (v/v). Two final ethanol precipitations in acetic acid and NET buffer (150 mM NaCl, 15 mM Tris-HCl pH 7.5, 1 mM EDTA) were successively performed. After washing in 70% ethanol, pellets were resuspended in 20 μL of sterile deionized diethyl pyrocarbonate-treated water and titrated by spectrophotometric measure of the absorbance at 260 nm.

### RT-PCR and quantitative PCR

1μg of total RNA was subjected to reverse transcription, during 1 hour at 37°C, using 1 μg of nonspecific hexameric random primers dN, 1mM dNTP, 10 mM dithiothreitol, 24 units RNaseOUT and 200 units of M-MLV-RT enzyme (Invitrogen). The PCR amplification was performed on 1:10 (v/v) of the 1:10-diluted reverse transcription reaction using 0.2 mM dNTP, 2.5 mM MgCl_2_ and 1 unit of Thermostart Taq DNA polymerase (Thermo Scientific), and 25 pmol of each specific primer :

NTS (5'-CAGCTCCTGGAGTCTGTGCT-3' and 5'-GAGTATGTAGGGCCTTCTGGG-3')'
NTSR1 (-5'-CGTGGAGCTGTACAACTTCA-3 and 5'-CAGCCAGCAGACCACAAAGG-3)
HER3 (5'-ATGGGGAACCTTGAGATTGTGCT-3' and 5'-ACAGCTTCTGCCATTGTCCT-3')
EGFR (5'-TTTCGATACCCAGGACCAAGCCACAGCAGC-3' and 5' AATATTCTTGCTGGATGCGTTTCTGTA-3')
HER2 (5'-GTGCTAGACAATGGAGACC-3' and 5'-CACAAAATCGTGTCCTGGTAGC-3')
18S (5'-AGGAATTGACGGAAGGGCAC-3' and 5'-GTGCAGCCCCGGACATCTAAG-3')
36B4 (5'-GTGCAGCCCCGGACATCTAAG-3' and 5'-GATTGGCTACCCAACTGTTG-3')

Semi-quantitative amplification was performed in a DNA thermal cycler 9700 (Perkin Elmer Applied Biosystem), and Maxima SYBRGreen qPCR Master Mix (Fermentas) in a Mx3000P qPCR system (Stratagene) was used for quantitative PCR.

### Patients

We studied 499 specimens of patients operated for Breast cancer in 2008 in Algeria. The average of patient age was 51.1 ± 11.7 years. The size of the tumor was 36.3 ± 21.7 mm. The SBR histopronostic grading was 6% grade I, 63% grade II and 31% grade III. The Subtypes were distributed as followed : 75.9% Invasive ductal carcinomas, 10% invasive lobular carcinomas, 2.2% Mucinous carcinomas, 1.20% Invasive papillary carcinomas, 1.6% invasive micropapillary carcinomas, 0.40% Invasive metaplasic carcinomas, 0.60% metaplasic carcinomas, 3.4%, invasive ductal and lobular carcinomas, 1.20% mixte Mucinous and ductal carcinomas, 3% mixte ductal and metaplasic carcinomas and 0.4% others. For TMA Three sample per specimen were analyzed.

### Immunohistochemistry

Deparaffinized TMA sections (4 μm) were subjected to heat-induced epitope retrieval in citrate buffer (pH 6.0). The sections were labeled for the target proteins using the avidin-biotin-peroxidase complex method. The slides were incubated at 4°C overnight with primary antibody included anti-NTS (1:200, SC-20806, Santa Cruz biotechnology®), and anti-ErbB3 (1:50, NCL-c-erbB-3, Novocastra™), anti-NTSR1(1:100; SC-7596, Santa Cruz Biotechnology®) and Anti-ErbB2 (1:400, A0485, Dako) was incubated at room temperature for 1hour and 30 minutes respectively. These slides were then incubated with appropriate biotinylated secondary antibodies, NTS (Trekkie Biotinylates rabbit link, Biocare medical®), NTSR1 (Biotinylated anti-goat IgG, Vector laboratories, Inc), ErbB3 (Trekkie Biotinylates mouse link, Biocare medical®). The antigen-antibody complex was revealed with avidin-biotinperoxidase complex, according to the manufacturer's instructions, NTSR1 (Vectastain ABC Kit, Vector laboratories, Inc.), NTS and ErbB3 (Trekavidin-HPR label, Biocare medical®). ErbB2 was biotinylated and revealed with the NovoLink™ Polymer Detection System (Leica®). NTSR1 and ErbB2 staining were done with diamino-benzidine tetrahydrochlorid, NTS and ErbB3 were done with aminoethyl carbazole. All slides were counterstained with hematoxylin then scored by an anatomopathologist (NM, FPL, or AC). The levels of staining were scored based on staining intensity within the tumor sample, with weak as 1, moderate as 2, strong as 3.

### Immunofluorescence assay

Cells were seeded on 12 mm-diameter glass slides for 24 hours, fixed in 5% paraformaldehyde for 1 hour at room temperature, permeabilized in PBS /0.5% Triton X-100 for 30 min and saturated for 20 min in PBS+ (1:100 (m/v) BSA, 1:250 (v/v) cold fish skin gelatin in PBS 1X, pH 8.0). Cells were then incubated overnight at 4°C with the primary antibody diluted to 1:100 in PBS 0.1% Triton X-100. NTS immunoreactivity was detected using a rabbit polyclonal anti-NTS immunoglobulin (NA1230, Tebu-Bio) and NTSR1 with a goat polyclonal antibody directed against the human COOH terminus of the receptor (C20, Santa Cruz Biotechnology). Slides were incubated for 1 hour with a fluorescent secondary antibody (1:100): a cyanin3 anti-rabbit immunoglobulin or a FITC-coupled anti-rabbit or goat immunoglobulin (Jackson ImmunoResearch). Nuclei were counterstained for 5 min with DAPI 1:50000.

## SUPPLEMENTARY FIGURES AND METHODS



## ABBREVIATIONS

NTS: neurotensin
NTSR1: neurotensin receptor 1
EGF: Epidermal growth factor
HERs: epidermal receptors
EGFR: Epidermal growth factor receptor
MMP: metalloproteinase
NRG: Neuregulin
Hb-EGF: Heparin-binding EGF-like growth factor
DCIS: ductal carcinoma in situ
OR: Odds ratio
