# Supplementary material for: Activation of EGFR, HER2 and HER3 by neurotensin/neurotensin receptor 1 renders breast tumors aggressive yet highly responsive to lapatinib and metformin in mice
Source: Oncotarget. 2014 Oct 3;5(18):8235–51. doi: 10.18632/oncotarget.1632 (PMC4226680; doi:10.18632/oncotarget.1632)
Supplement: Supplementary file 1 [file oncotarget-05-8235-s001.pdf]

SUPPLEMENTARY FIGURES AND METHODS

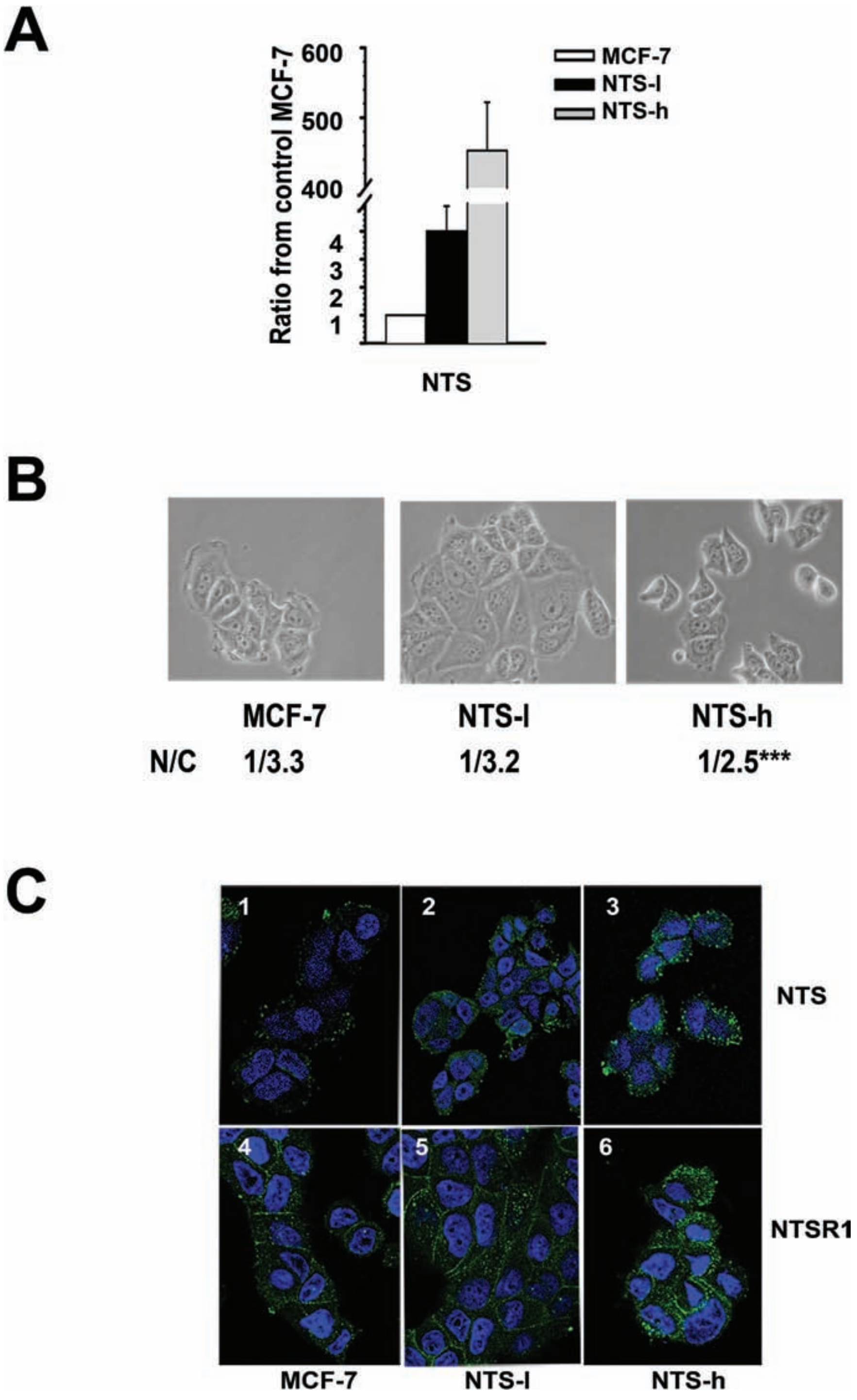

Supplementary Figure S1: Characterization of MCF-7 and MCF-7 overexpressing NTS cells. **(A)** Quantitative RT-PCR on MCF-7, NTS-h and NTS-l RNAs. Results represent the mean ± SEM of 7 independent experiments. **(B)** Typical cell images of MCF-7 cells (left), NTS-l (middle), NTS-h (left) acquired from an optical microscope at 400X magnification. Below, inserted nucleocytoplasmic ratio calculated on 100 cells. Student-Newman-Keuls Multiple Comparisons Test was performed on the data: ***$p<0.001$. **(C)** Typical immunocytochemistry labeling for NTS (top) and NTSR1 (bottom) in MCF-7, NTS-l, and NTS-h. Cells were seeded on glass slides, grown for 48h, and immunocytochemistry was performed as described in the Materials and Methods section.

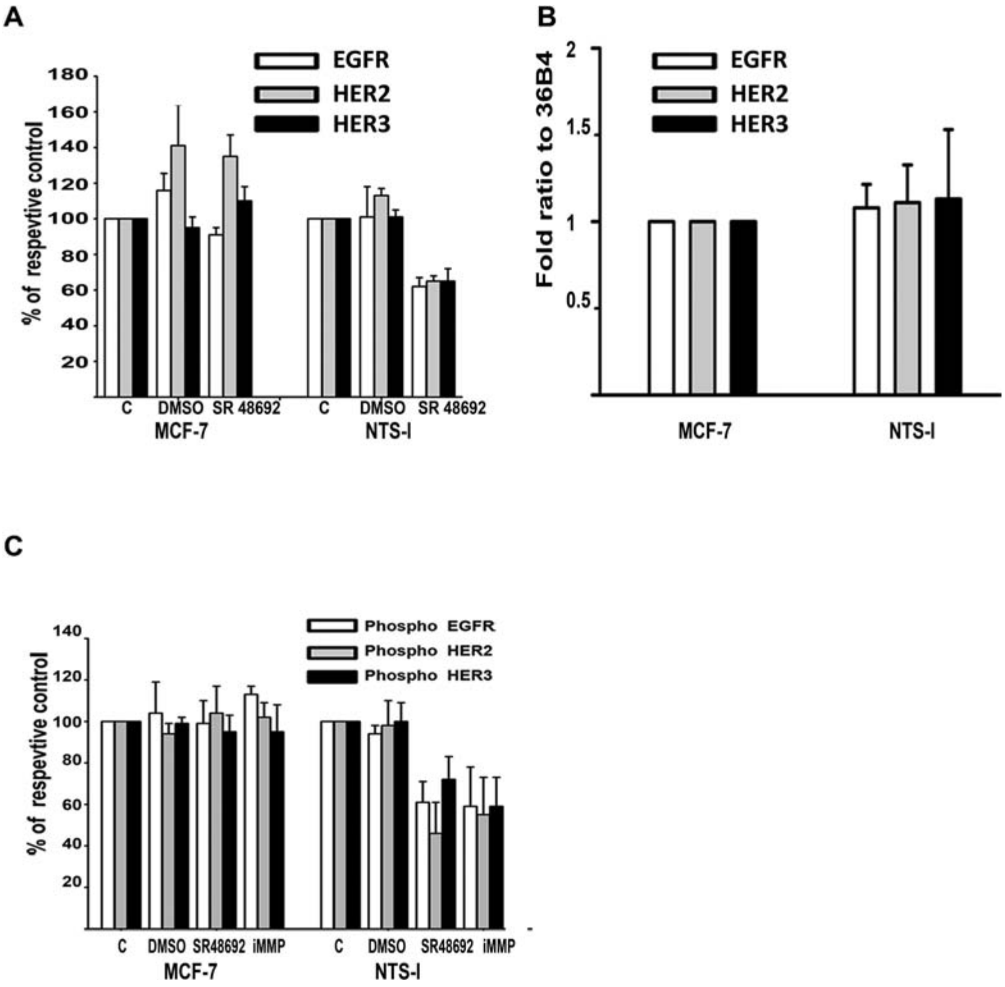

Supplementary Figure S2: Inhibition of EGFR, HER2, and HER3 basal expression and activation by NTSR1 antagonist. (A) Histograms representing intensity-based quantification of Western blot bands of basal total protein, EGFR, HER2, and HER3, control or treated with DMSO or 5x10^{-6} M SR 48692, in MCF-7 or NTS-l cells. Values are expressed as the percentage of the control MCF-7 cells and are the mean ± SEM of 4 to 7 independent experiments. **(B)** EGFR, HER2 and HER3 q-PCR performed on MCF-7, and NTS-l. **(C)** Breast cancer cells NTS-l or MCF-7, with the histograms representing intensity-based quantification of Western blot bands of phosphorylated protein, EGFR, HER2, and HER3, control or treated with DMSO, 5x10^{-6}M, SR 48692 or 25x10^{-9} M iMMP., Values are expressed as the percentage of the control MCF-7 cells and are the mean ± SEM of 3 to 5 independent experiments.

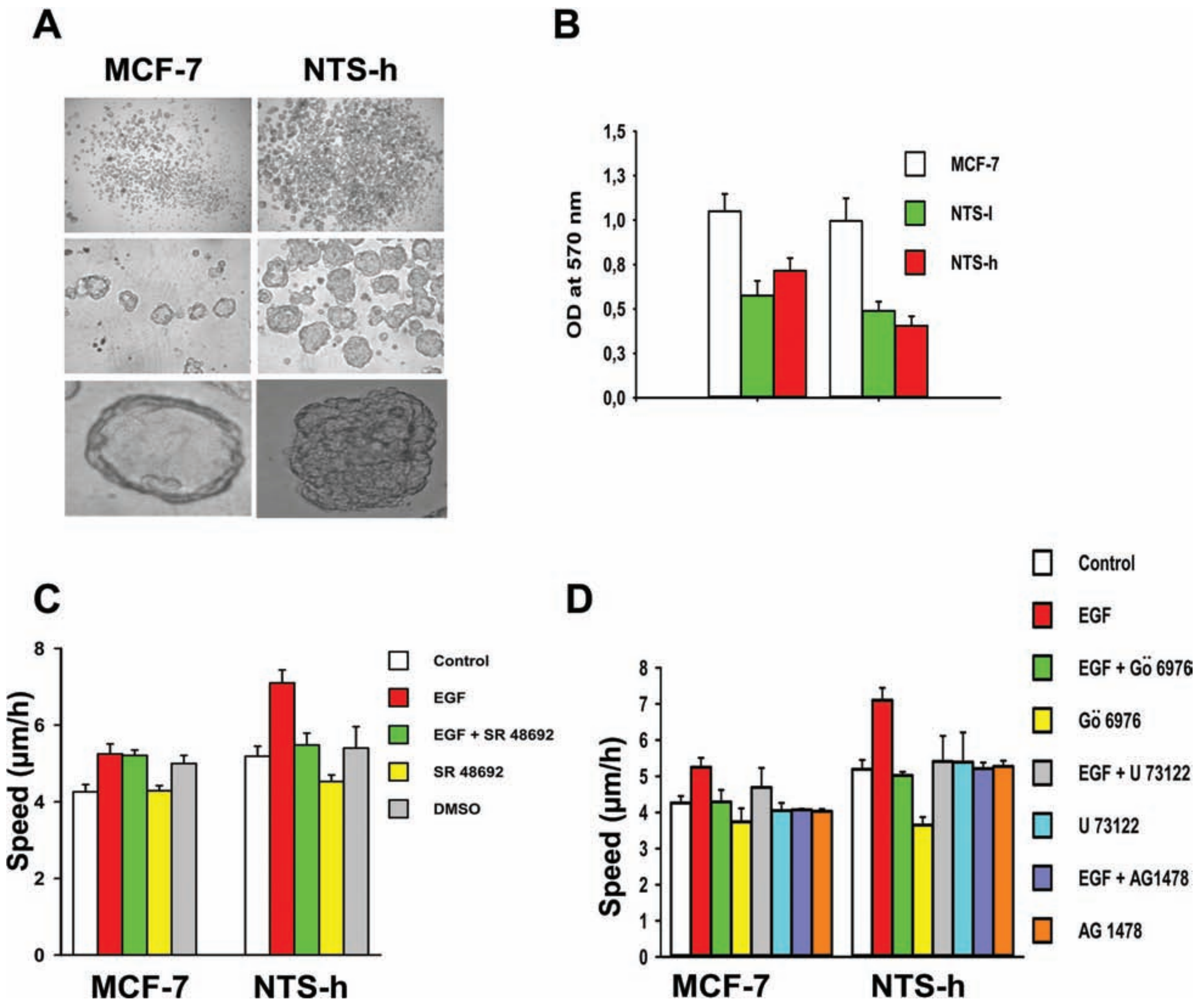

Supplementary Figure S3: NTS autocrine and paracrine regulation enhanced oncogenic cellular effects and EGF induced migration on matrigel matrix. **(A)** Representative pictures of 12 days cell colonies from MCF-7 (left) and NTS-h (right) at 50X (top), 100X (middle), 200X (bottom) magnification. **(B)** Adhesion assays were performed on matrigel supports. After 1h or 48h of seeding, cells were gently washed and the remaining attached cells were quantified by spectrophotometric analysis of crystal violet staining. Results represent the mean optic density ± SEM of 4 experiments. **(C)** Speed of migration on matrigel of MCF-7 and NTS-l cells control or treated with EGF (10 ng/mL), in the presence or not of SR 48692 ($5x10^{-6}$ M). Results represent the mean ± SEM of 9 to 10 independent experiments. **(D)** Speed of migration on matrigel of MCF-7 and NTS-l cells control or treated with EGF (10 ng/mL), in the presence or not of Gö6976 ($5x10^{-8}$M), U73122 ($5x10^{-6}$ M) or AG 1478 ($5x10^{-6}$ M). Results represent the mean ± SEM of 4 independent experiments. Results represent the mean ± SEM of 3 to 4 experiments. Student-Newman-Keuls Multiple Comparisons Test was performed on the data: ***P<0.001,**P<0.01, and *P<0.05.
